# *In Vitro* Coinfection and Replication of Classical Swine Fever Virus and Porcine Circovirus Type 2 in PK15 Cells

**DOI:** 10.1371/journal.pone.0139457

**Published:** 2015-10-02

**Authors:** Niu Zhou, Gang Xing, Jianwei Zhou, Yulan Jin, Cuiqin Liang, Jinyan Gu, Boli Hu, Min Liao, Qin Wang, Jiyong Zhou

**Affiliations:** 1 Key Laboratory of Animal Virology of Ministry of Agriculture, Zhejiang University, Hangzhou, PR China; 2 College of Veterinary Medicine, Nanjing Agricultural University, Nanjing, PR China; 3 China Institute of Veterinary Drug and Control, Beijing, PR China; 4 State Key Laboratory and Collaborative Innovation Center for Diagnosis and Treatment of Infectious Diseases, The First Affiliated Hospital, Zhejiang University, Hangzhou, PR China; College of Veterinary Medicine, CHINA

## Abstract

Increasing clinical lines of evidence have shown the coinfection/superinfection of porcine circovirus type 2 (PCV2) and classical swine fever virus (CSFV). Here, we investigated whether PCV2 and CSFV could infect the same cell productively by constructing an *in vitro* coinfection model. Our results indicated that PCV2-free PK15 cells but not ST cells were more sensitive to PCV2, and the PK15 cell line could stably harbor replicating CSFV (PK15-CSFV cells) with a high infection rate. Confocal and super-resolution microscopic analysis showed that PCV2 and CSFV colocalized in the same PK15-CSFV cell, and the CSFV E2 protein translocated from the cytoplasm to the nucleus in PK15-CSFV cells infected with PCV2. Moreover, PCV2-CSFV dual-positive cells increased gradually in PK15-CSFV cells in a PCV2 dose-dependent manner. In PK15-CSFV cells, PCV2 replicated well, and the production of PCV2 progeny was not influenced by CSFV infection. However, CSFV reproduction decreased in a PCV2 dose-dependent manner. In addition, cellular apoptosis was not strengthened in PK15-CSFV cells infected with PCV2 in comparison with PCV2-infected PK15 cells. Moreover, using this coinfection model we further demonstrated PCV2-induced apoptosis might contribute to the impairment of CSFV HCLV strain replication in coinfected cells. Taken together, our results demonstrate for the first time the coinfection/superinfection of PCV2 and CSFV within the same cell, providing an *in vitro* model to facilitate further investigation of the underlying mechanism of CSFV and PCV2 coinfection.

## Introduction

Virus coinfection or superinfection, a simultaneous or consecutive infection, has become a common phenomenon which involves the infection of the same type virus, a closely related virus or different virus species. For the past few years, some novel reassortant influenza A viruses were observed to sequentially assemble when a different subtype of avian flu virus, such as H7N9 [1, 2], H10N8 [3], H17N10 [4] and H18N11 [5] was coinfected or coexisted in individuals and populations. Moreover, infection of not only homologous viruses but also heterologous viruses was found to form hybrid viruses [6–9] or result in persistent infections [10] and interference between viruses [11–18]. Recently, porcine circovirus type 2 (PCV2), an immunosuppressive virus, was found persistently to be co-infected with other viruses, such as classical swine fever virus (CSFV) [19, 20], porcine reproductive and respiratory syndrome virus (PRRSV) [21–24] and porcine parvovirus (PPV) [25–28]. Undoubtedly, coinfection and superinfection are potential serious threats to public health and animal husbandry.

CSFV, a notifiable virus to the World Organization for Animal Health, is a small, enveloped virus with a non-segmented, single-stranded positive RNA genome and belongs to the genus *Pestivirus* of the family *Flaviviridae*. PCV2, which lies in the genus *Circovirus* of the family *Circoviridae*, is non-enveloped and contains an ambisense, single-stranded, closed-circular DNA genome [29, 30]. Prior clinical studies have reported that PCV2 infection interfered with the protective efficacy of an attenuated CSFV vaccine, whether applied simultaneously or consecutively [19, 20]. Recent research has also shown that PCV2 could decrease the infection and replication of an attenuated CSFV in primary porcine alveolar macrophages [20]. However, why and how PCV2 as a DNA virus interferes with replication of CSFV as an RNA virus is unknown due to the lack of an appropriate coinfection/superinfection model system. Thereby, we aimed to establish a coinfection system of CSFV and PCV2 *in vitro*, and provide an efficient way to study the potential underlying mechanism for CSFV and PCV2 coinfection and other virus coinfection, as well.

The present study explores an *in vitro* coinfection system of CSFV and PCV2 in a cell model. We found that the PK15 cell line could harbor high levels of replicating CSFV (PK15-CSFV cells). Furthermore, PK15-CSFV cells could be coinfected with PCV2 with a high replication rate and support the entire life cycle of both viruses. In this model system, PCV2 could replicate well, and the production of PCV2 progeny was not influenced in PK15-CSFV cells. However, reproduction of CSFV was impaired in a PCV2 dose-dependent manner. The coinfection of PCV2 and CSFV did not enhance cellular apoptosis in PK15-CSFV cells. Our study provides a cell model for deeper investigations of the collaborative pathogenesis of PCV2 and CSFV coinfection.

## Materials and Methods

### Cells, viruses and antibodies

PCV-free porcine kidney epithelial (PK15) cells [31] and swine testicle (ST) cells kept in our laboratory were maintained in minimal essential medium (MEM) (Gibco, Carlsbad, CA), and porcine alveolar macrophages 3D4/31 cells (CRL–2844, ATCC, Rockville, MD) [32] in RPMI–1640 medium (1640) (Gibco), supplemented with 10% Co–60 radiated fetal bovine serum (FBS) (Gibco) respectively. The PCV2 strain HZ0201 (AY188355, 10^6.4^ TCID_50_/0.1ml), isolated from a pig with naturally occurring postweaning multisystemic wasting syndrome (PMWS), was propagated in PK15 cells [33]. The polyclonal antibodies (pAb) and monoclonal antibody (mAb) against PCV2 Cap protein were prepared in our laboratory [34]. The attenuated lapinized Chinese strain of CSFV (HCLV strain, 10^4.0^ TCID_50_/0.1ml) was kept in our laboratory, and mAb WH303 to CSFV E2 protein [35] was kindly gifted by Prof. Trevor Drew of Animal and Plant Health Agency (former Veterinary Laboratories Agency), Weybridge, UK. Tandem dye pairs for super-solution microscopy, Cy^TM^ 3/Alexa Fluor^®^ 647-conjugated donkey anti-rabbit IgG and Alexa Fluor^®^ 405/Alexa Fluor^®^ 647-conjugated donkey anti-mouse IgG, were kindly gifted by Hangjun Wu from Core Facilities of Zhejiang University School of Medicine, Hangzhou, China.

### Inoculation with PCV2 and CSFV in cell lines

PK15, ST and 3D4/31 cell lines were used to propagate virus progenies. PCV2 was directly inoculated into cell subculture medium at the indicated multiplicity of infection (MOI) after the cells were adhesive, while CSFV was added at different MOIs when the cells monolayers had grown to 80–90% confluency and were washed gently three times with culture media before infection. Cells infected with viruses were kept at 37°C with 5% CO_2_ for 2 h, and culture media were replaced with fresh complete growth media containing 10% FBS for PCV2 and maintenance media containing 3% FBS for CSFV. At 72 h post-infection (hpi), cells were fixed to detect the infection rate by indirect immunofluorescence assay or collected for total RNA extraction to examine the virus mRNA level. Both cells and culture supernatants were subjected to three successive freeze-thawed to obtain the virus stocks for subsequent experiment use.

### Indirect immunofluorescence assay (IFA), confocal laser scanning microscopy (CLSM) and super-resolution microscopy (SRM)

The inoculation procedure and IFA protocol were performed as previously described [31]. Briefly, cells were seeded in 96-well plates at 2.0 × 10^4^ cells/well and the infection of PCV2 or CSFV as described above. After incubation at 37°C for 72 h, cells were washed twice with phosphate-buffered saline (PBS), fixed with a methanol-acetone mixture (1:1, v/v) at -20°C for 20 min and then blocked with PBS with 5% skimmed milk at 37°C for 1 h. For IFA, the cells were incubated with mouse mAb against PCV2 Cap or mouse mAb against CSFV E2 for 1.5 h at 37°C, followed by incubation with FITC-conjugated goat anti-mouse IgG [Kirkegaard & Perry Laboratories Inc. (KPL), Gaithersburg, MD] for 1 h at 37°C. The stained cells were observed using an IX71 inverted fluorescence microscope (Olympus, Tokyo, Japan). The infection status of virus in cell lines were determined by counting numbers of positive cells using ImageJ software.

For CLSM and SRM, cells grown on glass bottom dishes (In Vitro Scientific, Sunnyvale, CA) were inoculated with viruses for the indicated time, washed, fixed, permeabilized with 0.2% Triton X–100 in PBS and incubated at 4°C with primary antibodies overnight. The primary antibodies used included swine anti-Cap pAb and mouse anti-E2 mAb WH303. After being washed gently with PBS for five times, the cells were then incubated with FITC-conjugated goat anti-swine IgG (KPL) and Alexa Fluor^®^ 546-conjugated goat anti-mouse IgG (Invitrogen, Carlsbad, CA) as secondary antibodies at 37°C for 1 h. Cellular nuclei were stained with 1 μg/ml 4′-6-diamidino-2-phenylindole (DAPI) (Roche, Mannheim, Germany) at room temperature for 5 min, and then cells viewed with a LSM780 confocal laser scanning microscope (Zeiss, Oberkochen, Germany). For four-color CLSM, cells were labeled with a terminal deoxynucleotidyl transferase-mediated dUTP nick end labeling (TUNEL) kit (described below), inocubated with a rabbit anti-Cap pAb and a mouse anti-E2 mAb WH303 as primary antibodies and then inoculated with Alexa Fluor^®^ 546-conjugated donkey anti-rabbit IgG (Invitrogen) and CF^TM^ 647-conjugated donkey anti-mouse IgG (Sigma-Aldrich, St. Louis, MO) as secondary antibodies. For SRM, cells were inoculated with rabbit anti-Cap pAb and mouse anti-E2 mAb WH303 as primary antibodies as well, followed by tandem dye pairs, Cy^TM^ 3/Alexa Fluor^®^ 647-conjugated donkey anti-rabbit IgG and Alexa Fluor^®^ 405/Alexa Fluor^®^ 647-conjugated donkey anti-mouse IgG, as secondary antibodies. Cells were viewed with the N-stochastic optical reconstruction microscopy system (N-STORM) (Nikon, Tokyo, Japan).

### Titer determination of virus stocks

TCID_50_ values were introduced to detect infectivity of progeny viruses in virus stocks obtained as mentioned above. The titrations of PCV2 and CSFV were carried on PK15 and ST cells, respectively. The inoculation procedure for serially diluted virus stocks and IFA protocol were described above. The titers were determined by viewing the infected cells under a Olympus fluorescent microscope and calculated using the Reed-Muench method [36].

Copies of the viral genome in virus stocks were measured by absolute quantitative real-time PCR. For PCV2, total virus DNA was extracted from 100 μl of the stock samples by using the UNlQ–10 column virus genomic DNA isolation kit (Sangon Biotech Co., Ltd., Shanghai, China) following the manufacturer’s instructions. Sense (5’ GGTAACGCCTCCTTGGATACGTCAT 3’) and anti-sense (5’ CGCTTCTTCCATTCTTCTTGC 3’) primers were used to amplify a 136-bp fragment in PCV2 genome, including the full length of PCV2 stem-loop structure, which was cloned into the pMD™18-T vector (TaKaRa, Dalian, China) as a standard curve template for PCV2.

For CSFV, the viral RNA genome in the virus stocks was prepared with TRIzol reagent (Invitrogen) according to the manufacturer’s instructions. Reverse transcription of RNA was carried out using the Super Script First-Strand Synthesis System (Fermentas, Pittsburgh, PA) based on the manufacturer’s protocol. Sense (5’ CGACTGTCCATTGTGGGTTAC 3’) and anti-sense (5’ GGATTCTGGTGGTTTATTCTTGTT 3’) primers were used to amplify a 253-bp fragment between the N^pro^ gene and the C gene of CSFV, which was cloned into pMD™18-T vector as a standard curve template for CSFV. The viral genomes were assayed in triplicate by real-time PCR using the SYBR Premix Ex Taq (TaKaRa) in an ABI 7500 sequence detector system (Applied Biosystems, Carlsbad, CA). The virus copy number for each sample was calculated as the mean value of triplicate reactions.

To detect CSFV mRNA levels in cells, total celluar RNA samples were extract with TRIzol reagent. The next steps for relative quantitative real-time PCR were the same as that described above, except the β-actin gene was used as an internal standard for comparison of mRNA levels in cells instead of preparing a standard curve template. The sense (5’ TCATCACCATCGGCAACG 3’) and anti-sense (5’ TTGAAGGTGGTCTCGTGGAT 3’) primers for β-actin amplified a 100-bp fragment.

### Establishment of PK15 and ST harboring replicating CSFV

PK15 cells and ST cells were individualized with 0.5% Trypsin-EDTA (Gibco) and adjusted to 2.0 × 10^5^ cells/ml in MEM with 10% FBS. After the cells grew to 80–90% confluency, CSFV was added as described above. About 48 hpi, when cells infected with CSFV were fully confluent and serially subcultured. Passaged cells were maintained in MEM with 10% FBS for 72 h and then fixed for detecting proportions of CSFV-infected cells by flow cytometry or freeze-thawed to determine copies and TCID_50_ of virus stocks.

### Flow cytometry

After being individualized with 0.5% Trypsin-EDTA, washed and resuspended in PBS at the indicated time, the cells were fixed with 75% ethanol at -20°C for 20 min, rehydrated in PBS at room temperature for 5 min and then permeabilized with 0.05% Triton X–100 in PBS at 4°C for 20 min. The cells were incubated in PBS with 10% normal bovine serum followed by the mAb WH303 for 30 min at 4°C. Meanwhile, irrelevant antibodies served as background controls. Subsequently, the cells were incubated with a secondary antibody FITC-conjugated goat anti-mouse IgG at 1:500 dilution for 30 min at 4°C. All samples were placed on a shaker and protected from light. Thereafter, the antibody-labeled cells were washed three times with PBS and analyzed with an FC500 flow cytometer (Beckman, Brea, CA). Acquisition of > 5,000 events was performed, and data analysis was conducted using CXP software (Beckman).

### Cell proliferation assay

Cell proliferation activity was assayed by Cell Counting Kit–8 (CCK–8) (Beyotime Institute of biotech, Nantong, China) according to the manufacturer’s instructions. Briefly, cells in 96-well plates (2.0 × 10^3^ cells/well) were incubated in 100 μl of medium for various time points. At the end of each incubation period, cells in test wells were incubated with 10 μl CCK–8 at 37°C for 1 h in the dark. Wells containing medium without cells were assayed as background. The absorbance measured at 450 nm using an enzyme-linked immunosorbent assay reader (Bio Tek Instruments, Winooski, VT) was directly proportional to the activity of living culture cells.

### TUNEL assay

Apoptotic cells were detected using the TUNEL BrightGreen Apoptosis Detection Kit (Vazyme Biotech Co., Nanjing, China) to label the 3’-end of fragmented DNA according to the manufacturer’s instructions. The FITC-labeled TUNEL-positive and DAPI-labeled cells were imaged under a fluorescent microscope. After several (3 or more) fields were randomly selected from each sample, numbers of apoptotic cells and total cells were counted using ImageJ software. Ratios of TUNEL-positive cells to the total cells were calculated.

### Luminescent caspase–3/7 activation assay

Cells in a 96-well plate were inoculated with PCV2 at the indicated MOI. At 72 hpi, cells were gently mixed with Caspase-Glo^®^ 3/7 reagent (Promega, Madison, WI) and incubated for 1 h in the dark at room temperature. The culture supernatants were then transferred to a white opaque 96-well plate. Culture medium without cells served as the background control. The enzymatic activity of caspase–3/7 was measured using a SpectraMax M5 plate reader (Molecular Devices, Sunnyvale, CA).

### Inactivation of PCV2 with BPL

PCV2 inactivation was carried out by adding BPL (Yuanye Biological Technology co., Shanghai, China) into the virus stocks to a final concentration of 0.5 ‰, inocubating at 4°C for 36 h and decomposing BPL at 37°C for 2 h.

### Inoculation of PCV2 components

PCV2 proteins and genome were added to individualized and adherent cells at the final concentration of 1 μg/ml. At 72 h later, cells were fixed for analysis by the TUNEL assay or freeze-thawed to obtain virus stocks. PCV2 proteins were generated in prokaryotic expression systems with (His-Rep, His-ORF3, and His-ORF4) or without (Cap deleted the nucleus localization signal, dCap) a His-taq and kept in our lab. The PCV2 genome was extracted from concentrated PCV2 stocks and kept in our lab.

### Statistical analysis

Data were statistically analysed and graphed using GraphPad Prism 6 (GraphPad Software, San Diego, CA). Statistically significant differences between groups were determined using the Student *t* test or ANOVA. A *P* value of less than 0.05 was considered statistically significant.

### Ethics statement

Production of pAb and mAb in animals was approved by the Institutional Animal Care and Use Committee (IACUC) of Zhejiang University (Permit No. SYXK 2012–0178). All animal experimental procedures were performed in full accordance with the Regulations for the Administration of Affairs Concerning Experimental Animals approved by the State Council of PR China.

## Results

### Selection of sensitive cell lines exposed to PCV2 and CSFV

**Fig 1:**
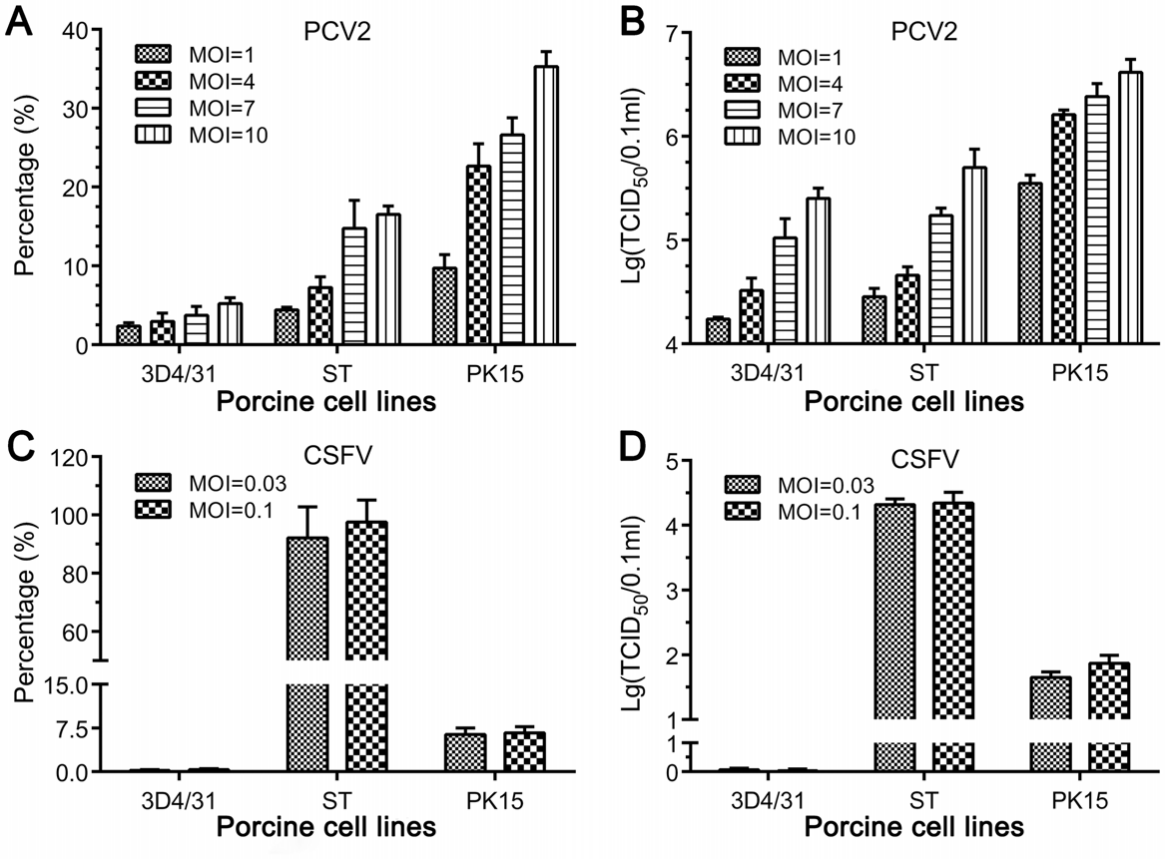
Infectivity of PCV2 and CSFV in different porcine cell lines. 3D4/31, ST and PK15 cell lines were inoculated with PCV2 at MOI = 1, 4, 7 and 10 or with CSFV at MOI = 0.03 and 0.1, respectively. After culture for 72 h, cells were fixed and stained for IFA, and then four fields were randomly chosen to count the percentage of the positive cells. Meanwhile, the cells were freeze-thawed to obtain virus stocks for determining virus titers. The percentage of PCV2-postive cells (A), PCV2 titer (B), percentage of CSFV-positive cells (C) and CSFV titer (D) were determined in 3D4/31, ST and PK15 cells.

In order to select a cell line sensitive to infection with PCV2 and CSFV, three widely used porcine cell lines PK15, ST and 3D4/31 were inoculated with PCV2 or CSFV at different MOIs. After the cells were infected with PCV2 at the MOIs, average percentages of PCV2-positive cells were counted by selecting four random fields of view of the tested cells, and progeny virus titers were determined in PK15 cells. The results indicated that all three porcine cell lines could be infected with PCV2 (Fig 1A and 1B). However, the susceptibility of different cell lines to infection varied. The PK15 cell line had the highest virus-positive cell percentages and progeny virus titers in stocks, compared with those of the ST and 3D4/31 cell lines. Moreover, the percentage of PCV2-positive cells and titer of progeny virus increased with the increase of MOI in different cell lines. These results showed that the PK15 cell line was the most permissive for PCV2 and could be infected in a PCV2 dose-dependent manner.

The susceptibility of cells lines PK15, ST and 3D4/31 to CSFV infection was determined by inoculating with the CSFV HCLV strain at two different MOIs (0.03 and 0.1), and titers of progeny virus were tested correspondingly in ST cells. As shown in Fig 1C and 1D, the low percentages of CSFV-positive cells and titers of CSFV in 3D4/31 cells indicated that they could barely be infected with CSFV. The PK15 cell line showed a moderate level of permissivity for CSFV, but the infection rate was still unsatisfactory. Among the three cell lines tested, ST was the most permissive for CSFV infection. However, CSFV-positive cells and progeny virus titers were not correlated with the amount of virus inoculum, indicating that the permissivity of these cell lines and not the original amount of virus affected the CSFV infection rate.

### Establishment of PK15 cell line harboring replicating CSFV

**Fig 2:**
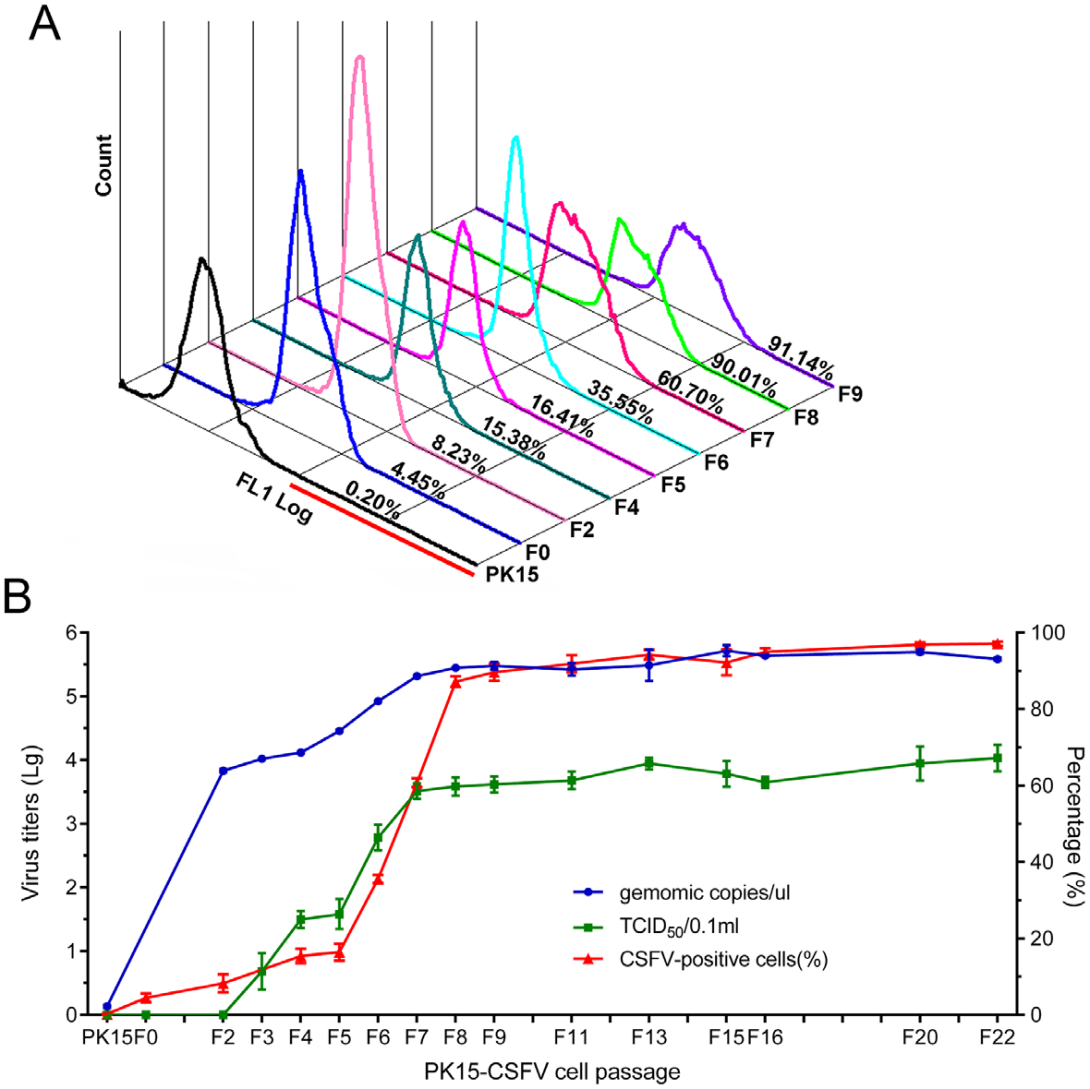
Viral infection status of CSFV in PK15 cell line harboring replicating CSFV. PK15 cells were infected with CSFV and then serially subcultured. Different passages of PK15-CSFV harboring replicating CSFV were collected to detect the viral infection status. (A) Histogram and proportion of CSFV-positive cells by flow cytometry. The red line indicates the positive gate for the cell population. (B) Three indexes of viral infection of PK15-CSFV cells: proportion of CSFV-positive cells using flow cytometry, viral genomic copies in virus stocks by absolute quantitative real-time PCR and infectivity of virus stocks by measurement of TCID_50_. Data are represented as means ± SD (n = 3).

**Fig 3:**
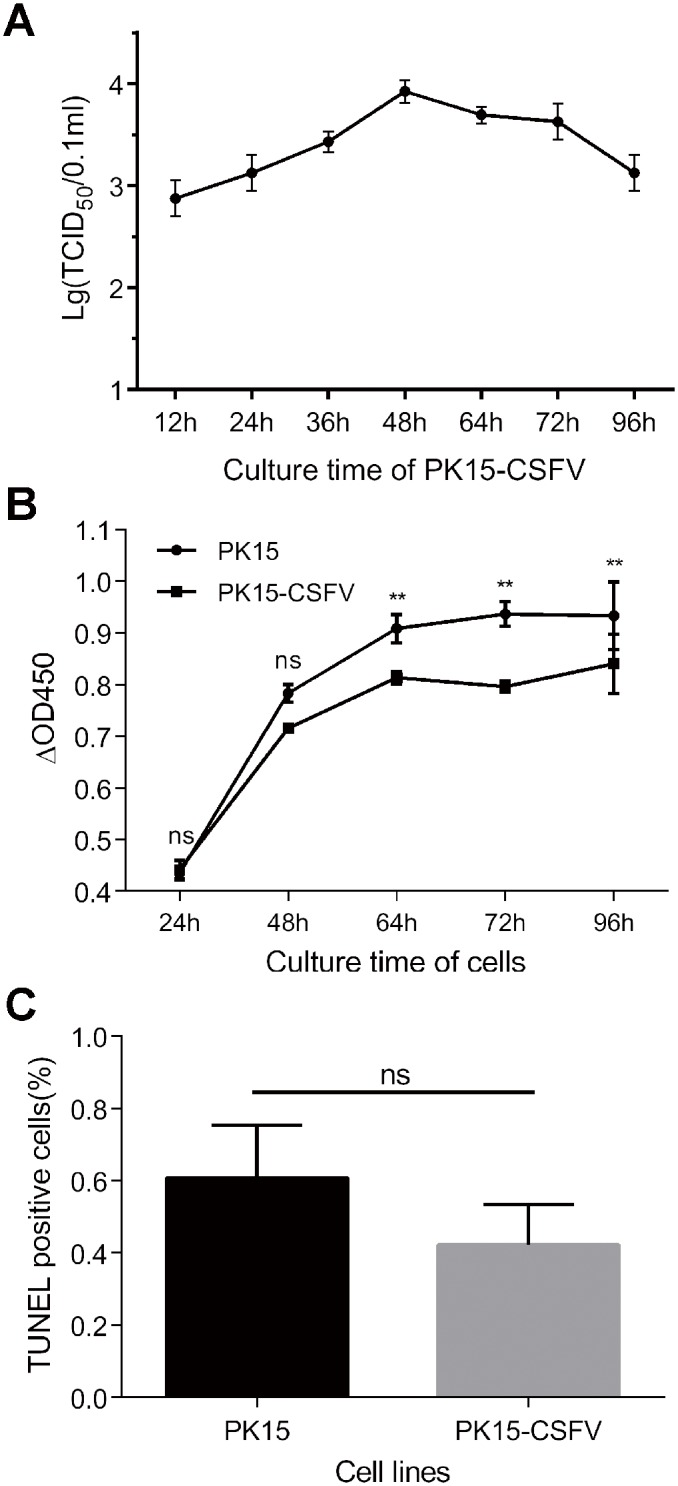
Biological characteristics of PK15-CSFV cells. PK15-CSFV cells were seeded into 6- or 96-well plates and cultured for indicated time points to detect the virus titer, cell viability and apoptosis. (A) Growth curve of CSFV in PK15-CSFV cells. (B) Viability of PK15-CSFV cells by CCK–8. (C) Proportion of TUNEL-positive cells at 72 h. Data are represented as means ± SD (n = 3 or 5; ns, *P* > 0.05; *P < 0.05; **P < 0.01).

Based on the results above, PK15 and ST cells were selected to establish cell lines harboring replicating CSFV. Cell monolayers at 80–90% confluency were inoculated with the CSFV HCLV strain at the MOI of 0.03 and subcultured at 48 hpi. CSFV-inoculated PK15 cells were designated PK15-CSFV and recorded as F0, while the first set of subcultured cells was labeled as F1, and so forth. PK15-CSFV cells were selected randomly after subculture for 72 h to detect CSFV-positive cells and virus titers. Flow cytometric analysis (Fig 2A) indicated that the percentages of CSFV infected cells gradually increased by serial passaging. As shown in Fig 2B, the indexes of CSFV infection increased gradually at the initial passages (F0 –F7). However, percentages of positive cells, genomic copies and TCID_50_ of CSFV in PK15-CSFV cells from the 8th passage were maintained at levels of more than 90%, 5 × 10^5^ copies/μl and 10^−3.6^/0.1 ml, respectively. Virus growth curves showed that CSFV titers increased gradually with the increase in culture time, reached a peak at 48 h (Fig 3A) and remained at a relatively stable level. Thus, the results indicated that CSFV maintained a high infection rate since the 8th passage in PK15-CSFV cells. Similar results were revealed in the ST cell line harboring replicating CSFV (ST-CSFV cells), which reached a nearly 90% positive rate and maintained a stable infection level (10^6.26^ copies/μl and of 10^4.1^ TCID_50_/0.1 ml) as early as the 2nd passage (data not shown).

To compare proliferation rates, PK15-CSFV cells and parental PK15 cells were seeded into 96-well plates at the same density, and then the CCK–8 assay was carried out after different time periods (Fig 3B). The results showed that after being cultured for 24 h and 48 h, the proliferation activities of PK15 and PK15-CSFV cells showed no difference. As time went on, the cell proliferation activities started to differ significantly (*P* < 0.01) at 64 h, reached a peak and remained at a stable level at subsequent time points. The TUNEL assay was carried out to determine if CSFV replication would cause any damage to the cells. As shown in Fig 3C, only cell apoptosis was not significant in PK15-CSFV cells as compared with PK15 cells (*P* > 0.05), indicating that the replicating CSFV did not change the normal characteristics of those cells.

### PCV2 and CSFV can productively infect the same cell

**Fig 4:**
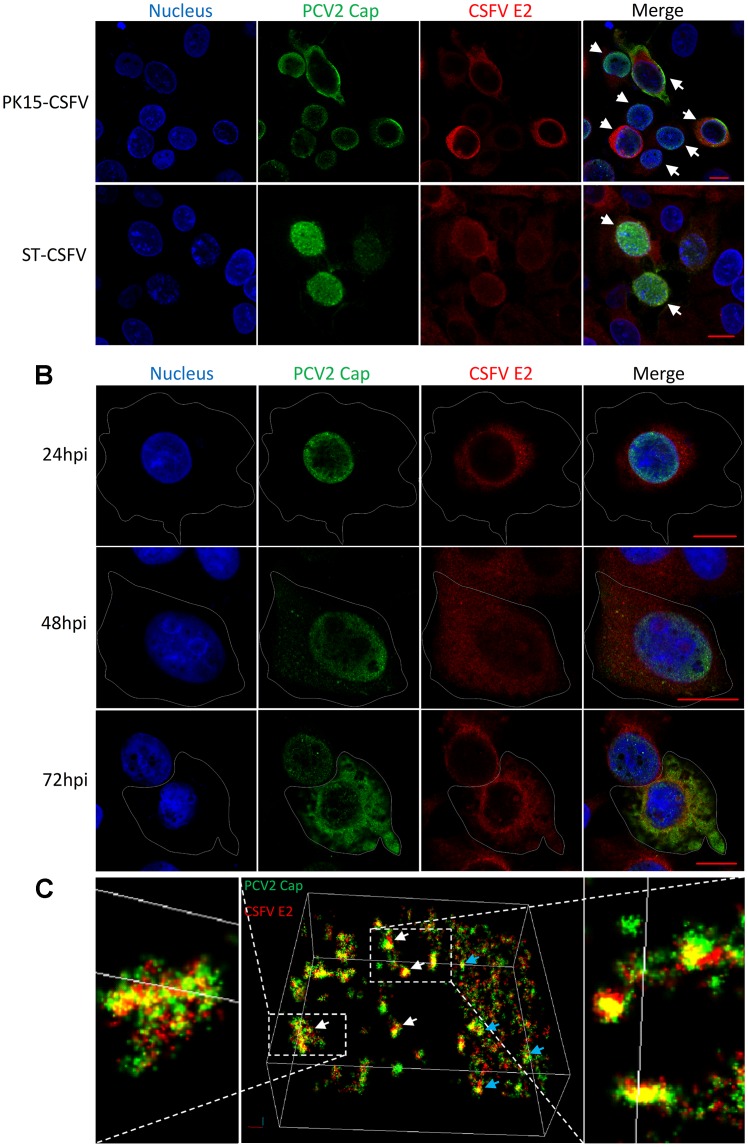
Subcellular localization of viral proteins in PK15 cells coinfected with PCV2 and CSFV. PK15-CSFV cells were seeded on glass bottom dishes and inoculated with PCV2 at MOI = 10. At indicated time points after PCV2 infection, cells were fixed and immunostained for PCV2 Cap (green) and CSFV E2 protein (red) as well as DAPI-stained for the nucleus (blue) and observed using CLSM and super-resolution microscopy. The red bar in the merged image represents 10 μm. (A) Overview of PCV2 infection at 72 hpi in PK15-CSFV and ST-CSFV cells. White arrows indicate double-stained cells. (B) Dynamic subcellular localization of viral proteins of PCV2 and CSFV in PK15-CSFV cells. (C) Super-resolution microscopy of PCV2 and CSFV in cells. Cells were fixed and immunostained for N-STORM. Images were taken and reconstructed to obtain 3D models of colocalization of PCV2 Cap and CSFV E2 proteins in the nucleus (white arrows) and in the cytoplasm (blue arrows).

Immunofluorescence analyses were performed to assess whether CSFV and PCV2 can replicate in the same cell and whether PCV2 replication could affect the subcellular localization of CSFV proteins. PK15-CSFV cells were inoculated with PCV2 (MOI = 1) and then fixed after various time periods. The PCV2 Cap protein and CSFV E2 protein were labeled with the corresponding Ab to determine the subcellular localization of each virus. Observation by CLSM (Fig 4A) showed that in all randomly selected fields the CSFV and PCV2 signals could be detected simultaneously in the same PK15-CSFV (upper) and ST-CSFV (lower) cells, indicating that CSFV and PCV2 could coexist in or coinfect a cell. Dynamic analysis in PK15-CSFV cells (Fig 4B) revealed that the PCV2 Cap protein accumulated in the nucleus at 24 hpi, started to appear in the cytoplasm at 48 hpi and mainly localized in the cytoplasm with little remaining in the nucleus at 72 hpi. By contrast, the CSFV E2 protein was mainly distributed in the cytoplasm of PK15-CSFV at the early stage of PCV2 infection but not in the nucleus, similar to the observation of PCV2-free PK15-CSFV cells. Interestingly, at the middle and the late stage of PCV2 infection, a few CSFV E2 proteins started to appear in the nucleus. Super-resolution microscopy (Fig 4C) further showed that the distance between of the PCV2 Cap (green) and CSFV E2 (red) were relatively close (yellow) both in the nucleus (white arrows) and in the cytoplasm (blue arrows). These results indicated that an entire life cycle of PCV2 and CSFV could be completed within the same cells.

### Coinfection rate of PCV2 and CSFV in PK15-CSFV and ST-CSFV cells

**Fig 5:**
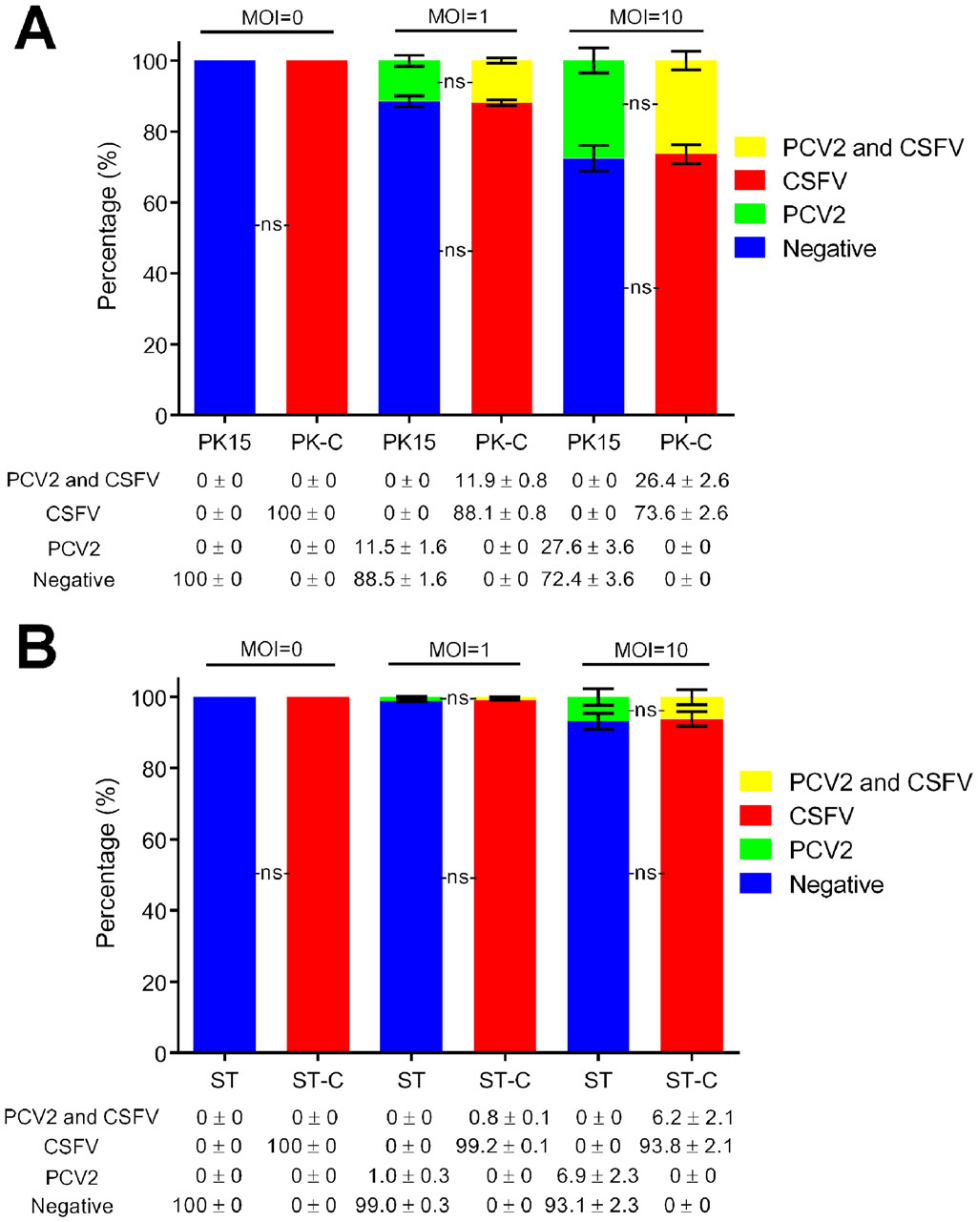
Coinfection rates of PCV2 and CSFV in PK15-CSFV and ST-CSFV cells. PK15-CSFV (A) and ST-CSFV (B) cells were adjusted to a density of 2.0 × 10^5^ cells/ml, inoculated with or without PCV2 at the MOI of 1 or 10 and then fixed for CLSM at 72 hpi. The infection rate of each virus and coinfection rate of PCV2 and CSFV were quantified by counting numbers of positive cells using ImageJ software. PK-C and ST-C represent PK15-CSFV cells and ST-CSFV cells, respectively. Data are represented as means ± SD (n = 4; ns, *P* > 0.05).

To investigate the coinfection efficiency of PCV2 in the same cell, cells were inoculated with PCV2 at the MOI of 1 or 10, and the rate of dual-positive cells for PCV2 and CSFV was quantified by counting the numbers of positive cells. As shown in Fig 5A, naïve PK15 cells were negative for both PCV2 and CSFV, and PK15-CSFV cells were negative for PCV2 and positive for CSFV. When inoculated with PCV2 at the MOI of 1, the rate of PCV2 positivity was 11.5 ± 1.6% in PK15 cells, while 11.9 ± 0.8% of PK15-CSFV cells were PCV2-CSFV dual-positive. After inoculation (MOI of 10), 27.6 ± 3.6% of PK15 cells were PCV2-positive cells and 26.4 ± 2.6% of PK15-CSFV cells were PCV2-CSFV dual-positive. The data indicated that the percentage of cells infected with PCV2 was dependent on the dose of virus inoculum in both PK15 and PK15-CSFV cells. Similar results also were observed in PCV2-infected ST and ST-CSFV cells (Fig 5B), albeit they had lower sensitivity to PCV2 infection in comparison with PK15 and PK15-CSFV cells. Interestingly, the difference in percentage of PCV2-positive cells and PCV2-CSFV dual-positive cells was insignificant between either PK15 and PK15-CSFV cells or ST and ST-CSFV cells (*P* > 0.05), indicating that the infection efficiency of PCV2 in PK15-CSFV cells was similar to that of PCV2 in PK15 cells. These data demonstrated that it was PK15-CSFV but not ST-CSFV cells that could ensure a high rate of dual infection and replication of PCV2 and CSFV.

### PCV2 infection decreases CSFV replication in PK15-CSFV cells

**Fig 6:**
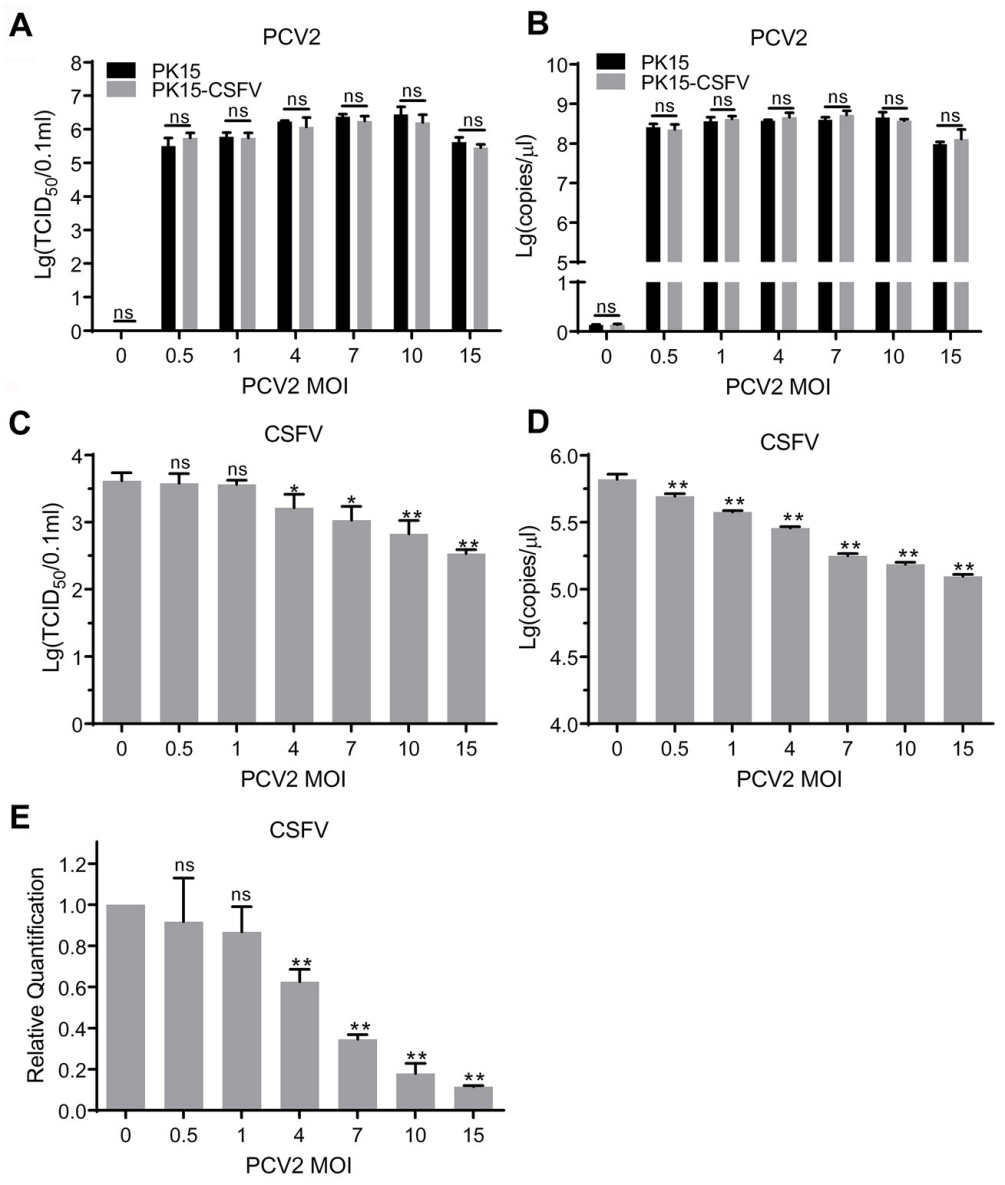
Replication of PCV2 and CSFV in PK15-CSFV cells. PK15 and PK15-CSFV cells were inoculated with PCV2 at MOI = 0.5, 1, 4, 7, 10 and 15. At 72 hpi, cells and supernatants were freeze-thawed to obtain virus stocks. Titers were determined by measurement of TCID_50_ in PK15 or ST cells and by absolute quantitative real-time PCR. (A) TCID_50_ of PCV2. (B) Genomic copies of PCV2. (C) TCID_50_ of CSFV. (D) Genomic copies of CSFV. (E) Total RNA of cells was extracted, and relative quantitative real-time PCR was used to detect the replication level of CSFV in PK15-CSFV cells infected with PCV2 at different MOIs. The ratio of CSFV mRNA to β-actin mRNA in mock-infected PK15-CSFV was defined as 1, and then the relative CSFV mRNA ratio in PCV2-infected PK15-CSFV cells was determined. Data are represented as means ± SD (n = 3; ns, *P* > 0.05; *P < 0.05; **P < 0.01).

Thus far, we had demonstrated that PCV2 could infect PK15-CSFV cells at the same efficient infection rate as in PK15 cells. To investigate if the PCV2 progeny replicated in PK15-CSFV was mature and infectious and to determine the ability of cells to harbor CSFV, PCV2 and CSFV titers were determined by measuring TCID_50_ and viral genomic copies in PK15-CSFV cells. As shown in Fig 6A and 6B, infectious PCV2 was efficiently produced in both PK15 and PK15-CSFV cells. No difference (*P* > 0.05) in titers of the PCV2 progeny was observed between PK15 and PK15-CSFV cells inoculated with PCV2 at the same MOI, indicating that PCV2 could replicate well in PK15 cells irrespective of the presence or absence of replicating CSFV. Therefore, we concluded that no significant exclusion of PCV2 occurred in its superinfection with CSFV.

However, as seen in Fig 6C and 6D, with the increase of PCV2 inoculum, TCID_50_ and genomic copies in stocks of progeny CSFV decreased gradually, and differences between PCV2-infected groups and mock-infected group (MOI = 0) gradually became more significant as well (*P* < 0.05 or *P* < 0.01). The mRNA analysis (Fig 6E) further showed that the CSFV transcripts decreased significantly (*P* < 0.05 or *P* < 0.01) in a PCV2 dose-dependent manner in PK15-CSFV cells. In particular, with PCV2 at the MOI of 15, the relative CSFV mRNA level reached as low as 0.115 ± 0.006% (*P* < 0.01), compared with that in PK15-CSFV cells without PCV2. This result confirmed that the reproduction of CSFV HCLV strain progeny was inhibited by PCV2 in PK15-CSFV cells and was dose-dependent.

### PCV2-induced apoptosis in PK15-CSFV cells

**Fig 7:**
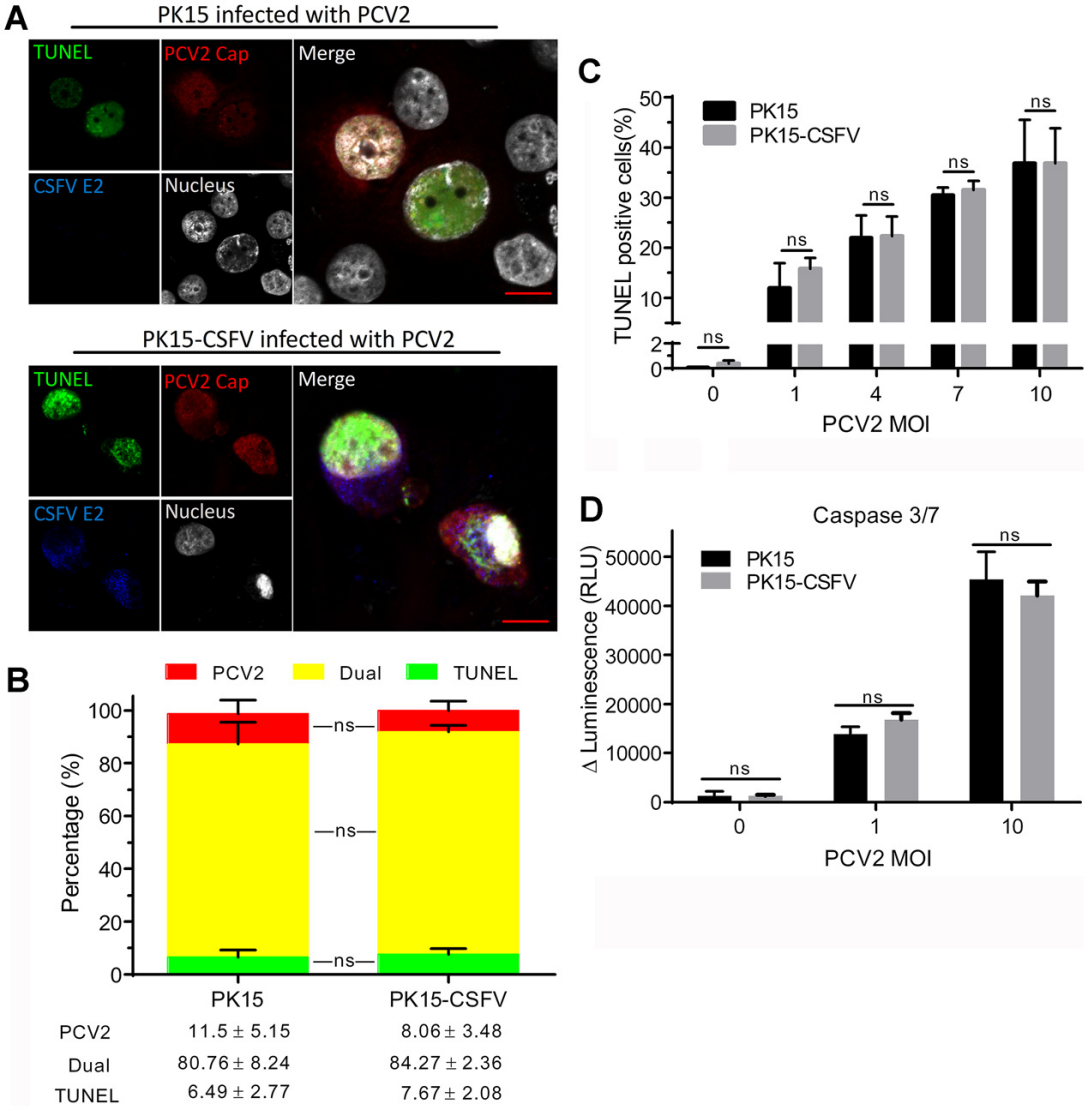
PCV2-induced apoptosis. (A) Cells infected with PCV2 (MOI = 1) were fixed for the TUNEL assay at 72 hpi and immunostained for viral proteins. Apoptotic cells (green), PCV2 Cap proteins (red), CSFV E2 proteins (blue) and nuclei (grey) are shown. (B) Statistical analysis of PCV2-induced apoptosis. Total numbers of TUNEL-positive, PCV2-positive and TUNEL-PCV2 dual-positive cells were counted, and proportions of each type of cells in all labeled (TUNEL or immunostained) cells were calculated in four random fields. (C and D) Cells inoculated with PCV2 of different MOIs were determined by TUNEL assay (C) and measurement of luminescent caspase–3/7 activities (D). The red bar in the merged image indicates 10 μm. Data are represented as means ± SD (n = 3 or more; ns, *P* > 0.05).

To further explore the possible mechanism by which PCV2 influenced CSFV replication, apoptosis was analyzed by the TUNEL assay and caspase 3/7 enzymatic activities were determined in PK15-CSFV cells with PCV2 infection. In both PK15 and PK15-CSFV cells infected with PCV2, the localization of PCV2-positive cells with TUNEL-positive cells was observed at 72 hpi of PCV2 infection (Fig 7A). The statistical analysis showed that the most predominant cells were TUNEL-PCV2 dual-positive cells in all labeled (TUNEL or immunostained) PK15 and PK15-CSFV cells after infection with PCV2, but the proportions of TUNEL-PCV2 dual-positive PK15 cells and PK15-CSFV cells were not significantly different (Fig 7B, *P* > 0.05). Subsequently, when cells were inoculated with PCV2 of MOIs, the TUNEL cells increased gradually in a dose-dependent manner (Fig 7C). Correspondingly, caspase 3/7 activities increased significantly in a PCV2 dose-dependent manner in PK15 and PK15-CSFV cells, but there showed no significant difference between PK15 cells and PK15-CSFV cells at the same MOI (Fig 7D, *P* > 0.05). These results showed that cellular apoptosis was only induced by PCV2 infection in PCV2-CSFV coinfected cells.

### Viral components of PCV2 do not contribute to cellular apoptosis in PK15-CSFV cells

**Fig 8:**
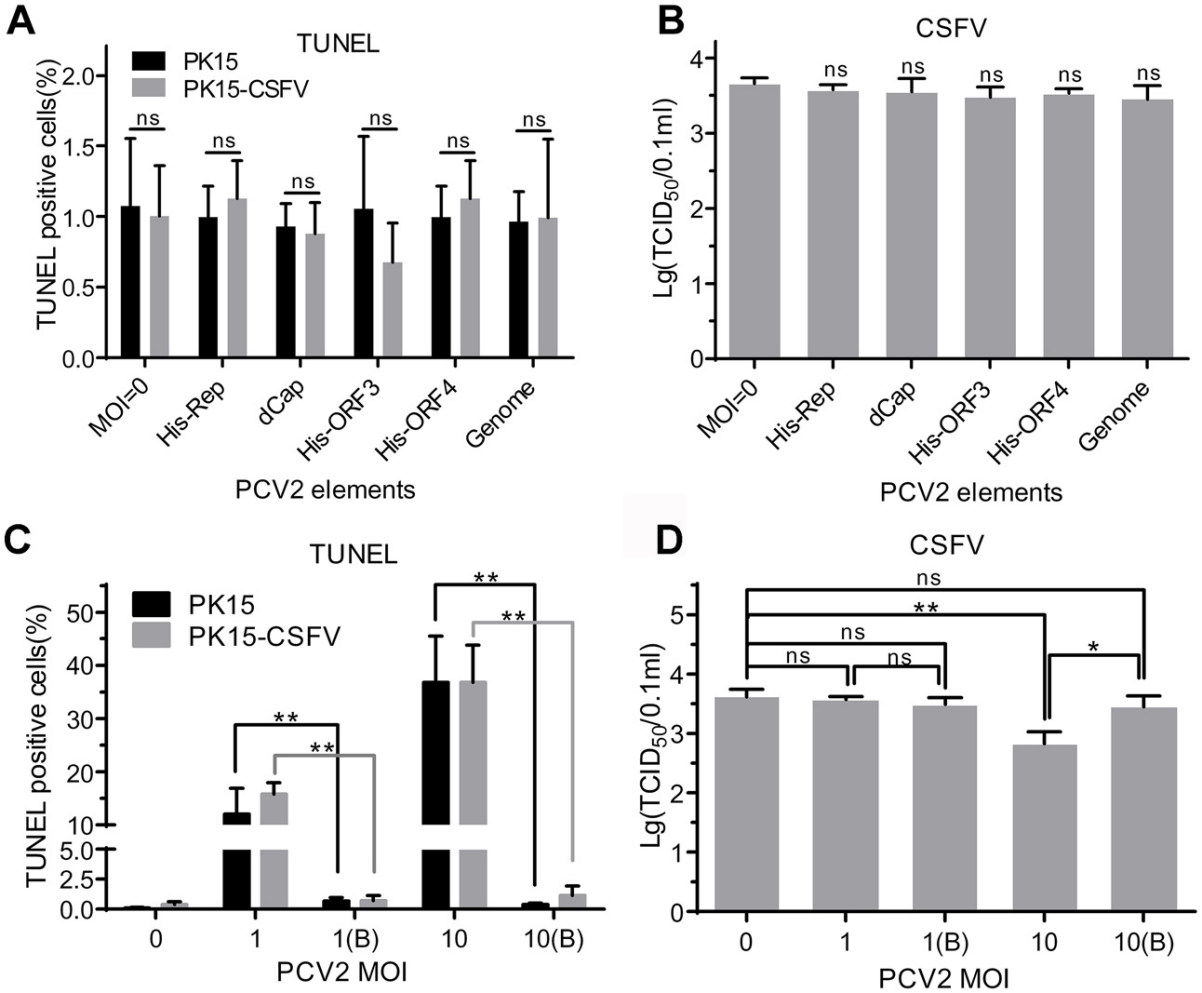
PCV2 but not PCV2 genome or PCV2-encoded components affects CSFV replication in PK15-CSFV cells. Cells were inoculated with PCV2 at MOI of 1 or 10 and pretreated with inactivated PCV2, genomic DNA of PCV2 or PCV2-encoded components. (A and C) Percentage of TUNEL-positive cells. (B and D) Titration of CSFV progeny at 1 (B) and 10 (B) MOI of BPL-inactivated PCV2. Data are represented as means ± SD (n = 3 or more; ns, *P* > 0.05; *P < 0.05; **P < 0.01).

To investigate whether PCV2-induced apoptosis involves viral components of PCV2, cells were treated with His-Rep, dCap, His-ORF3, His-ORF4 or genomic DNA of PCV2 and β-propiolactone (BPL)-inactivated PCV2, then the TUNEL assay and titration of CSFV were carried out. Data shown in Fig 8A and 8B revealed that none of the viral components of PCV2 tested caused apoptosis in PK15 or PK15-CSFV cells and did not affect CSFV replication. Similarly, PK15 and PK15-CSFV cells infected with the inactivated PCV2 could not further induce apoptosis and did not decrease the yield of CSFV in PK15-CSFV cells (MOI = 1 and 10) (Fig 8C and 8D). The results further confirmed that PCV2 replication but not viral components of PCV2 induced the apoptosis of PK15 and PK15-CSFV cells, and that the viral components of PCV2 did not interfere with the production of CSFV progeny.

## Discussion

Clinically PCV2 coinfection with viral pathogens is common in pig herds and becoming a greater concern [19–27, 37, 38], but a model for two or more viruses coinfecting or coexisting simultaneously within a single cell has not been constructed. The kidney has been reported as one of the major targets for replication of both PCV2 and CSFV *in vivo* [39–41], and 3D4/31 cells, developed from alveolar macrophages, support replication of CSFV [32] and PCV2 [42, 43]. In the present study, ST, 3D4/31 and PK15 cells were selected as host cells for constructing a model of CSFV and PCV2 coinfection/coexistence in a replicating context. PK15 cells were found to support the replication of PCV2 well but not that of CSFV, although a high CSFV titer was produced in ST cells (Fig 1). Additionally, the 3D4/31 cells could not support the efficient replication of PCV2 or CSFV. Therefore, considering that PCV2 could not propagate well in ST and 3D4/31 cells, the PK15 cell line in which CSFV could replicate well and stably (Figs 2 and 3) was chosen to construct an *in vitro* model system that allows for the coinfection/coexistence of both CSFV and PCV2.

Recently, PCV2 was demonstrated to be transported via direct interaction of the Cap protein with the cytoplasmic dynein IC1 subunit [44]. The presence of PCV2 Cap in the nucleus is indicative of PCV2 transcription [45], while PCV2 Cap in the cytoplasm suggests that whole progeny virus coated by Cap is forming and ready to be released [46]. In this study, dynamic subcellular localization analysis showed that PCV2 Cap proteins were located in the nucleus of PK15-CSFV cells at the early stage of PCV2 infection and in the cytoplasm of PK15-CSFV at the late stage of PCV2 infection (Fig 4B), indicating that an entire life cycle of PCV2 could be completed in cells harboring replicating CSFV. On the other hand, retention in the endoplasmic reticulum (ER) has been widely described for the E2 proteins of viruses within the family *Flaviviridae*, such as hepatitis C virus (HCV) [47] and bovine viral diarrhea virus (BVDV) [48]. During replication the E2 glycoprotein of CSFV has been found predominantly at intracytoplasmic membranes and only traces at plasma membranes [49, 50]. In our study, we demonstrated the colocalization of PCV2 Cap and CSFV E2 protein in coinfected cells (Fig 4C) and showed that PCV2 and CSFV could grow stably in coinfected PK15 cells (Fig 6). The results above indicated that PCV2 could replicate in the same PK15 cell harboring replicating CSFV. In addition, we observed that CSFV E2 was mainly diffusely distributed in the cytoplasm, however, gradually appeared in the nucleus as the PCV2 infection progressed (Fig 4B). The abnormal localization of E2 protein of CSFV HCLV strain possibly resulted from the coinfection with PCV2 in cells. However, determining whether PCV2 replication may induce the translocation of CSFV E2 protein from the cytoplasm to nucleus and defining the protein interactions between PCV2 and CSFV in coinfected cells will require further investigation.

Previous research showed that both hepatitis B virus and HCV could replicate in the same cell without overt interference, and specific inhibition of one virus did not affect the replication and gene expression of the other, arguing against superinfection exclusion (SE) [51]. Similarly, in this study we demonstrated that the entire life cycle of PCV2 was completed without interference or decrease in production in PK15 cells harboring replicating CSFV (Fig 6), indicating the absence of SE of PCV2 by CSFV. Conversely, PCV2 replication could decrease the replication of CSFV in a dose-dependent manner. SE has been observed for many viruses as a phenomenon in which an established virus infection interferes with or prevents a secondary virus infection, whether it is of the same, a closely related or even a different type [52]. Generally, it is restricted to homologous viruses and can occur at several stages during the viral life cycle, thus favoring entry of newly produced progenies into uninfected cells nearby and protecting a primary infecting virus from a competing virus [51]. Recently, a study of varicella-zoster virus and herpes simplex virus 1 displayed efficient SE in fibroblast cells and also in neurons with much lower efficiency [53]. Moreover, SE between homologous BVDV but not between heterologous vesicular stomatitis virus was observed to display possibly two mechanisms, one of which was at the viral entry level that required the E2 viral glycoprotein and the other at the viral RNA replication level [54]. However, the inhibition of CSFV replication by PCV2 in our study was different from those previous reports because PCV2 was inoculated in PK15-CSFV cells. Therefore, the mechanism for PCV2-mediated inhibition of CSFV replication in PK15-CSFV cells warrants further exploration in the future.

PCV2 has been shown to induce apoptosis by activating caspase–8 and caspase–3 pathways through the ORF3 viral protein *in vitro* [55], and ORF3 was found to play an important role in PCV2-induced apoptosis and pathogenesis *in vivo* [56]. CSFV was also reported to induce apoptosis via the 5’ and 3’ UTR of the genome of CSFV ALD strain and LPC strain [57, 58], whereas the N^pro^ of moderately virulent strain [59] and the NS2 of virulent Shimen strain [60] proteins could inhibit apoptosis. In our study, PCV2-induced apoptosis indeed appeared in PK15 and PK15-CSFV cells, and was dose-dependent (Figs 3, 7 and 8). However, this apoptosis showed no significant difference between PK15 and PK15-CSFV cells, although the inhibition of CSFV replication was positively correlated with PCV2-induced TUNEL-positive cells and activation of caspase 3/7. In addition, when the cells were pretreated with viral proteins and genomic DNA of PCV2, cellular apoptosis was not induced in PK15 and PK15-CSFV cells. These results indicated that the coinfection of PCV2 and CSFV did not enhance the apoptosis of infected cells.

In conclusion, we have determined two common cell lines which could be used as host cells for studies of CSFV and PCV2 coinfection. To obtain a higher coinfection rate and better understanding of CSFV and PCV2 coinfection, we established the cell lines PK15-CSFV and ST-CSFV which could stably harbor replicating CSFV in PK15 and ST cells, respectively, as demonstrated by flow cytometry and virus titering. The subcellular locations of PCV2 Cap protein and CSFV E2 protein in PK15-CSFV were detected, and we found that PCV2 could complete an entire lifecycle in PK15-CSFV cells and affect the localization of E2 slightly. The model system we established in PK15-CSFV cells ensured a high coinfection rate for both CSFV and PCV2 viruses and provided an efficient reference for other virus coinfection studies. We also demonstrated in this coinfection model system that PCV2 could replicate well and the production of PCV2 progeny was not influenced in PK15-CSFV cells, although generation of CSFV was impaired in a PCV2 dose-dependent manner. Further analysis indicated that PCV2-induced apoptosis in cells decreased the CSFV HCLV strain replication, which probably explains the serious clinical symptoms associated with CSFV and PCV2 coinfection and failure of the live-attenuated CSFV HCLV strain vaccine *in vivo*.
